# EXPLORING THE ROLE OF THE COMMUNITY AT THE END OF LIFE: EXPERIENCES FROM A SMALL COMMUNITY-LED END-OF-LIFE PROJECT

**DOI:** 10.1093/geroni/igad104.0390

**Published:** 2023-12-21

**Authors:** An-Sofie Smetcoren

**Affiliations:** Vrije Universiteit Brussel, Brussels, Brussels Hoofdstedelijk Gewest, Belgium

## Abstract

‘Care and living in community’ (CALICO) is an intergenerational co-housing and co-caring project in Brussels. Within this larger project, a small community-based initiative for palliative and end-of-life care was founded, called Maison Mourance. This initiative aims to mobilize capacity in the broader community to make ‘dying-in-place’ possible. Given the innovative and grassroots nature of Maison Mourance, the present study wants to understand its vision, functioning, roles of stakeholders involved and the challenges it faces. Therefore a qualitative study was conducted consisting of 10 in-depth interview with several stakeholders and 1 focus group with the steering committee. Their vision centers on creating a homelike environment where people at the end of their lives still feel part of the community. As organisation they want to be nonhierarchical in which each member, formal and informal, is valued and assigned a certain role. Attention is given to the central role of the patient and breaking taboos about dying and death by making it part of the (broader) community. Creating a supportive network of informal and formal actors in palliative care, and thus embedding care in the neighbourhood, is seen as a great added value. Future challenges are financing the project, expanding and keeping the network of volunteers motivated, designing an evaluation plan and selecting evaluation tools. Although this is a promising initiative in terms of community-led palliative care and for making ‘dying-in-place’ possible, the initiative faces several challenges and threats and thus it will be important to monitor future developments.

